# Network Diversity and Health Change among International Migrants in China: Evidence from Foreigners in Changchun

**DOI:** 10.3390/ijerph192316227

**Published:** 2022-12-04

**Authors:** Wenbin Wang, Yang Cao

**Affiliations:** Sociology Department, Jilin University, Changchun 130012, China

**Keywords:** health change, immigration network, international immigration in China, destination-country networks, home-country networks

## Abstract

Objective: Is the migration process likely to lead to sustained changes in individual social networks and health status? There are many controversies about the relationship between migrants’ networks and migrants’ health. An important reason may be that the constraints of specific social contexts on immigrant networks and health consequences are neglected. This study distinguished two types of social networks of international immigrants to China—their Chinese networks (Chinese-Net) and home-country networks (Motherland-Net). In addition, the study investigated the construction basis of immigrant social networks and health effects based on the Chinese context. Method: A cross-sectional survey was conducted in 2017, 2018, and 2019. The survey was carried out by an on-site questionnaire survey of foreigners in China in the entry-exit hall of the National Immigration Administration. The survey asked foreigners about their immigration experience, labor and employment, social networks, and access to health care. Results and conclusions: Immigrants from more developed countries are more likely to succeed in establishing Chinese-Net and reduce the dependence on Motherland-Net. The Japanese and South Korean immigrants tended to be associated with their home-country compatriots, excluding the Chinese from social contacts and immigration networks. The mixed residence of non-home-country immigrants reduces both the Motherland-Net and Chinese-Net of international immigrants. From the perspective of the health effect, the establishment and expansion of Chinese-Net did not present the “bright side” of encouraging immigrants to reach a better health status. In contrast, Motherland-Net has a stronger explanatory power for the health changes in immigrants.

## 1. Introduction

Driven by globalization and the adjustment of immigration policies, China, as a traditional migrant-sending country, is increasingly becoming the destination of many international migrants [1]. According to the seventh national census of the National Bureau of Statistics of China, there are approximately 850,000 foreigners living in the Chinese mainland, mainly coming to China for business, employment, and study (See http://www.gov.cn/xinwen/2021-05/11/content_5605792.htm (accessed on 30 September 2022); considering that illegal immigrants were not included, the number is likely to be underestimated). China is not a traditional country of immigrants, and the history of immigrants coming to China is relatively recent. Therefore, there are very few special studies on international immigrants in China, and the majority of these studies focus on African immigrants in Pearl River Delta, in south China (such as Guangzhou or Foshan) [2,3,4]. However, studies have pointed out that a broader group of immigrants in other Chinese cities exhibit different life patterns from the typical African immigrants in Pearl River Delta [5].

Many studies have shown that the link between migration networks and social adaptation is a key issue for migration research in all countries, but health consequences are often excluded. Migration is not only regarded as an economic process, but also as a social process, in which immigrant networks and embedded immigrant social capital play a continuous role. Migration networks are considered to positively affect the social adaptation of migrants, including increasing economic and educational opportunities, maintaining family integrity, and accelerating upward integration [6,7,8]. However, although the positive or negative impact of social networks and social interactions on health has been widely emphasized, its function in migrant networks is little investigated [9,10]. The drastic change in living environment often leads to the weakening of formal social support, leading to the deterioration of the health status of migrants compared with native residents [11], and the impact of their migration network on their health status is also important.

Recent studies have noted that migration networks are not always consistent or positive. Personal ties to immigrants in the home-country and the destination-country population may play completely different roles in immigrants’ subsequent survival and development [9,10,11]. For one, the different statuses of various individuals in the social context may lead them to develop immigrant networks with varying qualities and quantities of social capital [12,13]. Therefore, we should not ignore internal distribution differences and potential health consequences of migration networks.

As China has a limited history of receiving immigrants during the modern era, what influence the Chinese context might have on the social network and health effect of immigrants is largely unknown. Different from developed countries, most of which are traditional destinations for immigrants, and the south–north migration of traditional immigrant waves, China started its unique immigrant reception process only after entering the new century. First, thanks to its continuous rapid economic growth and its important position in the global division of labor, China continues to attract immigrants from both developing and developed countries, forming two new waves of migration: South–south migration and north–south migration. Second, due to its long cultural tradition and recent immigration history, the Chinese native culture represented by Confucianism is still in an absolutely dominant position, which may have different behavioral influences on immigrants with different cultural backgrounds and relations to it (such as countries inside and outside the Confucian cultural circle). Finally, China maintains a pragmatic and prudent immigration policy, expecting immigrants to contribute to social development, but also concerned about the risks of introducing large numbers of immigrants. Except for a few cities with developed international trade (such as Guangzhou and Yiwu), immigrants are dominated by international students and foreign employees of enterprises. They are allowed to come to China only after being strictly screened by local schools and enterprises and receiving an invitation. After coming to China, they usually live in a certain area under the arrangement and assistance of their schools or enterprises. This residential area contains not only immigrants of their own ethnic background, but also immigrants from other countries.

On the basis of the above characteristics, this study focused on the following two issues: 1. How will these factors, which are unique to immigrants in the Chinese context, affect their construction of immigrant networks? 2. On this basis, what are the impacts of various migration networks on immigrants’ health changes?

### 1.1. Health Change and Migration Networks

The social determinants of health model argue that a person’s health is the result of a combination of multiple factors. The first is personal characteristics and lifestyle (gender, age, race, etc.), the second is the macro-socioeconomic and cultural background, and the third is the community and social network of the individual [14,15,16,17]. When migrants leave their home society and enter a new and unfamiliar one, they undergo an adaptation process filled with health risks. The social determinants of migrants’ health, such as language and work skills, social attitudes, and cultural orientations, and the composition of social networks, all change with the process of migration, bringing new challenges and pressures to the health status of migrants. The very limited evidence from Chinese society also points to this challenge, African immigrants in Pearl River Delta often face significant barriers to access to health care and are not satisfied with the services they receive [18]. The migration process is one of the important driving forces leading to the change (usually deterioration) of the health status of individuals [19,20]. Although contrary results suggest that the health status of migrants may be better than local residents, this is generally regarded as being explained by the “healthy migrant” hypothesis: Only healthier people can withstand the health pressures of migration and complete the migration process [21].

As an intermediate mechanism in regard to migration and social adaptation, migration networks are often mentioned. However, there are many controversies about the relationship between migration networks and migrant health. Supporters of the migration network and cumulative causality theory assume that the migration network is a dynamic process connecting the origin and destination. Therefore, it promotes the continuous self-maintenance and diffusion of immigration waves [14]. However, how do migration networks affect the survival of migrants once they make it to their destination? In the positive view, social networks and social capital can help them access other resources, improve their social support and health knowledge, help them find a suitable job, reduce the burden of work and life, and provide emotional protection for immigrants to cope with the cultural pressure imposed by the destination society. Therefore, these factors would have a positive effect on the physical and mental health of migrants [22,23,24]. However, a small number of negative views suggest that social networks could also have a negative impact on health; for example, they could promote the spread of negative emotions among different immigrant groups [25,26]. Although not everyone agrees on how immigrant social networks affect health, they almost all agree that these networks are important for immigrant health, in conjunction with cultural, economic, and other factors. 

These studies tend to treat health and migration networks more as established outcomes than as changing ones [27]. While migrants inevitably face greater health risks when entering unfamiliar societies, they differ in how they respond to health challenges. After the completion of migration, immigrants will try to establish and maintain a stable reciprocal relationship with a new environment to help them avoid becoming victims in the new social and cultural system [28], in a process called social adaptation. Addressing health challenges is also an important part of this social adaptation process. However, subsequent studies have suggested that the adaptation process of immigrants is not linear, but full of selection and segregation, caused by incompatibilities between individual characteristics and social environment [29,30,31]. Since formal support is absent or unstable, individuals’ social behaviors are more dependent on informal social support, represented by social networks, which then have a greater impact on their health. The communication objects of immigrants are no longer limited to their relatives or fellow citizens, who are homogeneous and have a strong connection; instead, they will also involve residents and immigrants from other countries in the place of migration. The composition of the immigration network includes interpersonal contact with residents and other immigrants, which may lead to different health consequences [24]. In addition, the focus on the process of health change allows us to eliminate the “health migration” hypothesis and gain a better understanding of the health consequences of the migration process.

In the same way, not all social networks of immigrants can be viewed as constituting forms of social capital. Moreover, it is highlighted that networks and health among individuals are continuously evolving and self-adjusting, with high correlation to the social context and social structure [32]. There are two orientations to social capital based on their different levels: The collective level [33] and the individual level [34]. The individual level of social capital comprises the social resources contained in social networks, which determine the value of “capital.” Social network theory typically divides networks into strong and weak ties, with these different types of ties associated with different types of social capital. Bonding social capital is the closed social bond between people, characterized by connections among family members, close friends, neighbors, or members of the same ethnic group [35]. This social capital, based on close family and friendship bonds, serves the self-interest of individuals or small groups. In contrast, bridging social capital tends to be open and inclusive, covering people from different social groups and backgrounds, thus forming broader social connections and cohesion [36]. If bonding social capital is suitable for a satisfactory life, then bridging social capital should be crucial to helping individuals to become successful in life [35].

Using this distinction, in many cases, immigrant networks are only regarded as solid connections with relatives or people of the same nationality. Moreover, the social capital generated is closer to bonding social capital emphasized by Putnam [23,37]. International immigrants have more resources for social communication in “post-migration” social adaptation. In addition to expanding strong links and bonding social capital with ethnic groups in their home countries, it is possible to seek bridging social capital. This is more open and heterogeneous, functioning through weak connections with residents. The various types of immigrant networks, especially the different compositions of motherland immigrants and residents in the immigrant network will have varying effects on the health change in the immigrant network owner [24,25].

Migration network distribution and function in different environments and the crowd is not necessarily consistent [38]. For immigrants entering the Chinese context, they are likely to maintain ties with their native ethnic group as well as establish ties with Chinese residents. However, their different positions in the Chinese social context may drive them to make different choices, which will have an impact on their health change. Drawing on the research above, this study splits immigration networks into two: Social networks with native Chinese residents (Chinese-Net), more suitable for bridging social capital, and social networks with motherland migrant (Motherland-Net), more suitable for bonding social capital. In this study, in the Chinese context, the construction and influences of two different networks were investigated based on social structure.

### 1.2. International Migrants’ Network Construction in China

There are noticeable differences among various immigrant groups in China, which cannot be neglected when we examine international immigration in China. This study focused on the structural characteristics of immigrants in the context of Chinese society. It investigated three dimensions: Economic background, cultural concept, and mixed living pattern.

#### 1.2.1. Economic Background

The ethnic origins of immigrants are often different in various societies. In the Changchun sample, interviewees come from 143 different countries and regions, among which 84.15% are from developing countries, and 15.85% are from developed countries. Among developing countries, India (12.72%) and Pakistan (8.84%) had the highest proportion, whereas, among developed countries, South Korea (7.89%), the United States (2.54%), and Japan (1.34%) had the highest proportion. There is no “typical” ethnic group, with absolute superiority in number, and ethnic groups with different economic and cultural backgrounds are evenly distributed among international immigrants in Changchun. Due to China’s short history of welcoming immigrants, most international immigrants to China are first-generation immigrants; accordingly, they are still significantly influenced by their origin countries. 

The economic gap between the source and destination countries of immigrants is regarded as the basis for the generation of immigration waves by classical immigration theories, such as New Economics of Migration and Dual Labor Market theories, which entails that migrants will choose to migrate from their home country to a more developed country to spread family risks and gain financial rewards. However, these theories on international migration overwhelmingly rely on the South-to-North Flows [39], failure to assess migration consequences that may arise when the economic background of the source country is similar to or better than the destination country. Some evidence has shown that about a third of all international migratory flows occur between developing countries [40]; China’s important position in the global industrial division of labor has not only attracted immigrants from developing countries, but also became a work migration destination for junior staffs and senior managers of multinational enterprises in some developed countries.

Therefore, the link between the economic background of the source country and the construction of the migration network becomes more differentiated. In the initial stage of entering a foreign society, migrants generally need to build a home-country network through ethnic ties to obtain basic support [16]. Immigrants from more developed countries usually have better economic resources and human capital, which can help them in the achievement of greater value in the labor market. Due to their economic value, the receiving units then pay more attention and support to them, and they also find it easier to obtain the welcome of residents in Chinese society. Therefore, individuals’ needs for ethnic affiliation decrease, and this also reduces the ethnic norms that restrict their interactions [30]. Relatively speaking, immigrants from more developed countries are less dependent on the Motherland-Net, thus limiting the expansion of their Motherland-Net. Simultaneously, advantageous flow options from north to south and a more mature intermediary and support system help them take more initiative in contact with Chinese society, thus building a broader Chinese-Net. On this basis, the hypothesis regarding the economic background is proposed:

**Hypothesis** **1.** *The migration network of international immigrants in China varies due to the different economic backgrounds*.

**Hypothesis** **1.1.** *Compared with immigrants from developing countries, immigrants from more developed countries have a higher proportion of Chinese-Net*.

**Hypothesis** **1.2.** *Compared with immigrants from developing countries, immigrants from more developed countries have a lower proportion of Motherland-Net*.

#### 1.2.2. Cultural Concept

In addition to economic factors, the cultural concept perspective is often neglected by existing studies. The East Asian ethnic group is a particular cultural group appearing in Chinese society. According to the World Values Survey (WVS), Japanese, South Korean, and Chinese societies have similar cultural backgrounds. At the same time, they collectively constitute the Confucian cultural circle, which has certain characteristics that distinguish it from other cultures. The similarity and closeness of East Asian cultures also shows its strength in their social networks and social capital. Although the level of social capital varies widely across countries, social ties have been improved to be rather strong and networks rather closed in East Asia, which cause strong ties to be more preferred and more powerful. As a result, East Asia has markedly less bridging social capital than Western Europe or North America, for instance [41,42].

When Japanese and Korean immigrants, who also belong to East Asian societies, meet Chinese residents in a Chinese context, due to the similarity and closeness of cultural concepts, which characteristic will dominate the construction of the immigration network of Japanese and Korean immigrants? The question may hinge on whether Chinese residents are likely to be these migrants’ “own people”. If similarity dominates, Chinese, Japanese, and Korean residents are more familiar with each other’s customs, cultural concepts, and even languages based on the long history of cultural exchanges. This may help Japanese and South Korean immigrants to regard Chinese residents as more suitable friends and communicate more with Chinese residents [31] (Data from www.worldvaluessurvey.org (accessed on 30 September 2022). The countries and regions belonging to the “Confucian cultural circle” are Japan, South Korea, Mainland China, Taiwan, Macau, Mongolia and Hong Kong. With the exception of Japan and South Korea, the Changchun sample does not include immigrants from other regions and countries). In turn, they will be less dependent on ethnic ties and home-country networks.

However, it is also found that immigrant groups consisting of Chinese, Japanese, and Koreans tend to have stronger family and ethnic identities after entering a foreign society. This closeness may be helpful for school education and lead to economic success but will constrain individual social interaction choices [23,31]. For example, when East Asian ethnic groups seek partners, closed ethnic ties may play a more important role, driving them to seek partners from their own ethnic group and reject external partners. For Japanese and South Korean immigrants, although Chinese residents are similar to “us,” they are still “others” with whom we have no ethnic ties or strong ties. East Asian ethnic groups tend to maintain the Motherland-Net and reject the Chinese-Net. On the basis of previous research on the prevalence of social networks in East Asian ethnic groups, we hypothesize that closeness may play a more important role; therefore, the following hypothesis regarding the cultural concept is proposed:

**Hypothesis** **2.** *The migration networks of international immigrants in China vary due to different cultural concepts*.

**Hypothesis** **2.1.** *The proportion of Chinese-Net in East Asian ethnic groups is lower than non-East Asian ethnic groups*.

**Hypothesis** **2.2.** *Compared with non-East Asian ethnic groups, East Asian ethnic groups have a higher proportion of Motherland-Net*.

#### 1.2.3. Mixed Living Pattern

Residential assistance provided by receiving units may profoundly impact the network construction of international migrants. The international immigrants in Changchun are mainly technical personnel from enterprises and students at college. These embedded international migrants can receive close help from their units, including financial support, accommodation arrangements, and assistance with relevant procedures. Compared with African businesspeople in Pearl River Delta, who struggle alone and find it difficult to obtain formal social support, the help provided by receiving units makes up for the gap in social support for international immigrants in Changchun. It effectively reduces the difficulties international immigrants face in Changchun due to entering a foreign society. Moreover, the residence forms related to the receiving units are factors shaping the differences in the social networks of international immigrants in Changchun.

For safety and convenience, centralized residence is directly provided to international immigrants in Changchun as part of social support. For example, the university provides alternative student apartments for international students, and enterprises offer rental assistance for foreign technical personnel on the premise of respecting their wishes. The international immigrant settlement in Changchun is different from the spontaneous gathering of traditional immigrants from bottom to top; instead, it is realized in the centralized system arrangement from top to bottom. Under this institutional arrangement, the residential boundaries of international immigrants in different regions, skin colors, and languages are broken, and the heterogeneity and internationalization degree of international immigrant settlements are enhanced. The international immigrant settlements in Changchun are “international villages.” This mixed pattern may help international migrants break free from the strong constraints of their home-country networks, but it may also further isolate them from Chinese society and residents. On this basis, the hypothesis from the destination is proposed:

**Hypothesis** **3.** *The network construction of international immigrants in China is affected by the mixed form of the destination*.

**Hypothesis** **3.1.** *The more non-home-country immigrants live in Changchun, the lower the proportion of the Chinese-Net will be*.

**Hypothesis** **3.2.** *The more non-home-country immigrants live in Changchun, the lower the proportion of the Motherland-Net will be*.

### 1.3. Health Effects of Disparate Migration Networks

Regarding the heterogeneity of migration networks, it is worth considering whether different types of migration networks are conducive to the health change in international migrants in China. In particular, which social network is more likely to help immigrants to China deal with their apparent health challenges? In fact, the effects of both Chinese-Net and Motherland-Net on health are considered positive by most studies, although some studies believe that the two types of social networks have no direct relationship with health or have negative effects [43]. However, the influences of Chinese-Net and Motherland-Net on the health change in Chinese immigrants have never been compared. They follow different possible influence paths, and the resulting health consequences may also be different.

Theoretically, there are two potential pathways for the positive effects of social networks and social capital on health. One is the information and resource pathway. Social networks are helpful for individuals to obtain more information about health behaviors and a healthy life. When individuals are faced with medical service needs, the existence of their social network improves the accessibility of medical services and health facilities, and avoids the possibility of health damage as early as possible. The second path is the emotional and normative path. High level of social interaction and mutual trust in social relationships provide sufficient emotional support, which can reduce the impact of migration stress on physical and mental health. Trusting social group interactions can lead to a sense of mastery and responsibility for oneself and one’s group. Individuals are more likely to accept and follow group norms of behavior and curb unhealthy behaviors, such as smoking, alcohol, and substance abuse [44,45]. At the same time, group interaction may also attract individuals to participate in more health promotion or disease prevention behaviors, such as physical exercise.

The positive roles of Chinese-Net and Motherland-Net for health are present along different paths: Chinese-Net may be more conducive to the information and resources pathway, while Motherland-Net deals more with the emotional and normative pathway. Due to the large social and cultural differences between immigrants and local residents, the Chinese-Net usually contains more heterogeneous information and resources, which may be more favorable for international migrants to obtain necessary medical and health information. For example, when international migrants are sick and need treatment, if they have the help of Chinese friends, they can enjoy better medical services in Chinese society [5]. In contrast, homogeneity information from Motherland-Net may not help international immigrants improve their access to medical resources, but based on the tight-knit, trustful Motherland-Net, migrants receive a large amount of emotional support to cope with the psychological shock and loneliness of entering a strange society. Under the group norms of Motherland-Net, migrants are more likely to maintain their old lifestyles without interference, avoid self-marginalization under Chinese social pressures, and have more health-boosting opportunities to participate in group activities and physical exercise with home-country compatriots [44,46].

Considering the relatively good health status of Chinese international migrants and the stable support provided by their employers, we believe that Motherland-Net may be a more important factor affecting the health of Chinese international migrants than Chinese-Net. The effects of Motherland-Net and Chinese-Net on health changes are essentially a discussion of the resources and emotional dimensions behind health changes. Although health facilities providing international care are scarce and expensive [47], the majority of high-quality healthcare resources in China are concentrated in public hospitals and open to the whole society. Due to the relatively good health status of international migrants to China, their demand for medical resources is relatively low. Moreover, when there is a need for medical services, immigrants can also seek help from their units through formal channels, rather than relying on the information advantages provided by Chinese-Net. The influence of Chinese-Net on their health changes may also be limited. The emotional support and group norms provided by Motherland-Net, through prolonged contact in daily life, are difficult to replace for international migrants. Trust and dependence on Motherland-Net by international migrants may have a more sustained impact on their lifestyle and health behaviors. On this basis, the hypotheses of health change effects are proposed:

**Hypothesis** **4.** *Motherland-Net affects international migrants’ health change more than Chinese-Net*.

**Hypothesis** **4.1.** *Regardless of Chinese-Net value, the health of international migrants does not change significantly*.

**Hypothesis** **4.2.** *The higher the Motherland-Net, the healthier the international migrants become*.

## 2. Methods

### 2.1. Changchun Sample

On the basis of Changchun sample of Survey of Foreigners in China (SFRC), Changchun is used as a study sample to represent a new pattern of immigration composition in Chinese society. Compared with the Pearl River Delta samples, which have attracted more attention, the composition of Changchun samples is more diverse. Moreover, as Changchun is located in the geographic center of Northeast Asia, and has geo-cultural ties with Korea and Japan, the Japanese and Korean ethnic groups have become relatively distinct and important among Changchun foreigners. This makes it convenient to examine the influence of the economic background and cultural concept of immigrants.

In terms of location, due to the lack of sufficiently developed international trade in Changchun, the proportion of businesspeople among immigrants is lower and the proportion of international students and foreign employees of enterprises is higher. Immigrants are most commonly supported and assisted by local schools and enterprises, and a mixed living pattern is more typical. More importantly, with the improvement in the degree of opening up to the outside world and the internal transfer of industrial clusters, inland cities with a large number of people but less developed international trade have also began to attract immigrants. The Changchun sample, which is dominated by international students and corporate employees, although smaller in number, is likely to represent a more general pattern of immigration composition in Chinese society in the future: Immigrants present a diversified economic background and cultural concept from the source country, and form a centralized mixed residence mode with interracial immigrants with the help and support of schools and enterprises.

### 2.2. Data Sources

The data in this study were obtained from the Survey of Foreigners in China (SFRC), which was launched by the Institute of National Governance of Sun Yat-sen University in Guangzhou in 2016 and subsequently expanded to six cities, including Changchun, Hangzhou, Xi’an, Lanzhou, Yiwu, and Xuzhou. The author’s research team conducted the Changchun survey in September and October 2017, 2018, and 2019. The survey was carried out by an on-site questionnaire survey of foreigners in China in the entry-exit hall of the National Immigration Administration. Before the survey, relevant ethical approval and informed consent were obtained. Each participant voluntarily participated in the survey on the premise of being informed of the research objectives and data confidentiality policy regarding social-demographic characteristics of the participants, including gender, age, education level, and marital status. Moreover, the survey asked foreigners about their immigration experience, labor and employment, social networks, and access to health care. After questionnaire screening and data cleaning, 1624 samples were finally used for analysis, which is the largest sample size and the most extensive thematic survey data for international immigrants in Northeast China.

### 2.3. Main Variables

**Figure 1:**
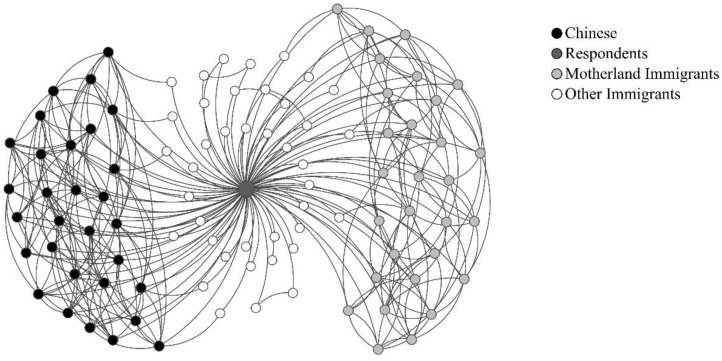
The ideal network structure for a foreigner in Changchun.

The dependent variables included migration network and health change. Two migration networks with different characteristics were generated based on the network scale. The opportunity structure of intergroup interactions is strongly influenced by the relative group size. The fewer the people of the same nationality, the less likely they are to have friends of the same nationality [31]. The variables above were generated by relative quantity rather than absolute quantity to avoid the influence of chance structure on network characteristics. First, we measured the number of Chinese residents, the number of people from their home countries, and the number of immigrants from other countries known by respondents in Changchun, as three continuous variables. Second, the total size of the network was obtained by adding the number of Chinese, motherland immigrants, and other immigrants. Finally, we divided the number of Chinese and the number of motherland immigrants by the total size of the network, respectively to obtain the Chinese-Net (mean = 0.306, STD = 0.260) and the Motherland-Net (mean = 0.307, STD = 0.246). The generated variable is a continuous proportional variable between 0 and 1. For explanation, we took the percentage and scaled it to produce a continuous variable from 0 to 100. As other migrants’ networks were usually recreational [48], they were not examined further here. Depending on the network size, an ideal network structure for a foreigner in Changchun might be similar to Figure 1.

Health change comes directly from the questions in the questionnaire. We asked participants to indicate changes in their health status after coming to China. Originally a five-level scale was used; we reduced it to “Same or became unhealthier than before” and “became healthier than before.”

Independent variables included economic background, cultural concept, and mixed living pattern. The Human Development Index (HDI) was used to assess the economic background of immigrants’ original country. This is a more comprehensive and nuanced assessment than considering developing vs. developed countries, with migrants classified as coming from high-HDI countries (higher than China), mid-HDI countries (similar to China), or low-HDI countries (lower than China). Then, immigrants from East Asian vs. non-East Asian groups (0 = immigrants from East Asia) were distinguished to measure the influence of cultural concepts. The variable of residence form was generated according to the distribution of non-home-country immigrants in the place of residence (0 = no or few non-home-country immigrants live in the place of residence).

In addition, we further controlled for survey years, age, sex, years of education, marital status, Chinese proficiency, residence duration, Engel’s coefficient, and motherland of immigrants in the place of residence. Meanwhile, in the health change model, we further controlled current self-rated health to better investigate the health change effect.

## 3. Results

Table 1 shows a multivariable linear regression model with Chinese-Net and Motherland-Net as dependent variables. We introduced variables related to economic background, cultural concept, and mixed living pattern, respectively.

Models 1 and 2 are the influencing factor models of Chinese-Net. The Chinese-Net of immigrants higher-HDI countries is significantly higher than the immigrants from lower-HDI countries, which supports Hypothesis 1.1. In comparison, the Chinese-Net of East Asian groups is significantly lower than the non-East Asian groups, which supports hypotheseis 2.1. Moreover, the Chinese-Net of international immigrants who live together with more immigrants from other countries is significantly lower, supporting Hypothesis 3.1.

Models 3 and 4 are the influencing factor models of the Motherland-Net. The results revealed that the Motherland-Net of immigrants from higher-HDI countries is significantly lower than the lower-HDI countries. The Motherland-Net of East Asian groups is significantly higher than the non-East Asian groups, supporting hypotheses 1.2 and 2.2. The Motherland-Net of international migrants, with more non-home migrants living in their residential areas, is significantly lower, supporting Hypothesis 3.2.

To verify the robustness of the above findings, in Table 2, we took the original ratio of Chinese-Net and Motherland-Net (i.e., the continuous ratio between 0 and 1 without percentage enlargement) as the dependent variable and constructed a proportional logistic regression model. The model settings are consistent with Table 1. The results revealed that the international immigrants from higher-HDI countries have significantly higher Chinese-Net and significantly lower Motherland-Net. For international migrants from East Asian groups, the proportion of Chinese-Net and Motherland-Net in their migration network both are significantly lower. When more non-home immigrants live in the same area, the proportion of Chinese-Net and Motherland-Net in their migration network is significantly lower. In summary, the data findings above are consistent with those in Table 1.

Note: 1. The coefficient is the odds ratio. Standard errors are robust standard errors; 2. To avoid very small coefficients, the original values are used here for Chinese-Net and Motherland-Net rather than the percentage values expanded to equal proportions. 3. *** *p* < 0.01, ** *p* < 0.05, * *p* < 0.1; 4. On the basis of Table 1, the control variables further add self-rated health.

**Figure 2:**
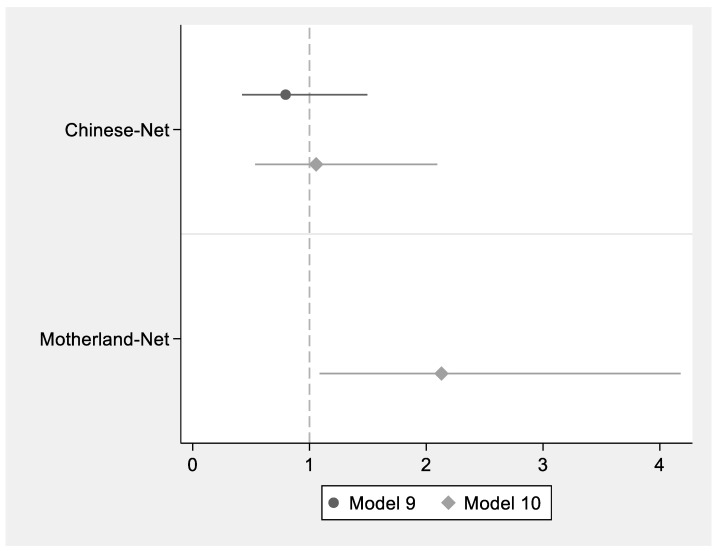
Logistic model for health change analysis of immigrant networks (N = 1624).

To compare different effects of Motherland-Net and Chinese-Net on immigrants’ health change, we constructed a logistic model of the impact of the two immigrant networks on health change, as shown in Figure 2.

The results in Model 9 indicated that the occurrence ratio of Chinese-Net on health change is less than 1 and that it is not significant. In Model 10, although the coefficient of Chinese-Net is greater than 1, it is still not significant. The impact of Chinese-Net on the health change in immigrants tends to be not very positive. In contrast, the impact of Motherland-Net on the health change in immigrants tends to be positive. In Model 10, it can be found that Motherland-Net has a significant positive influence on whether they have become healthier after entering China. Hypothesis 4 is supported by the data.

In summary, after considering the Chinese context characteristics of immigrants, we distinguished two different types of immigration networks, Chinese-Net and Motherland-Net, and constructed a model to investigate its health effectiveness from the perspective of the process of dynamic changes.

According to our data results, the economic background, cultural concept, and mixed residence pattern have a significant impact on the difference in the migration network of international immigrants in China. Specifically, compared with immigrants from less-developed countries, immigrants from more developed countries are more likely to succeed in establishing Chinese-Net with the advantages of economic background provided by their home countries, and reduce the dependence on ethnic ties and Motherland-Net. Despite cultural similarities with Chinese society, the preference of East Asian cultural concepts for strong ties and closet networks indicated that the Japanese and South Korean immigrants tended to be associated with their home-country compatriots, excluding the Chinese from social contacts and immigration networks. The mixed residence of non-home-country immigrants reduces the Motherland-Net of international immigrants and further blocks their contact with Chinese society, which is not conducive to the establishment of a Chinese-Net.

From the perspective of the health effect, the establishment and expansion of Chinese-Net did not present the “bright side” of encouraging immigrants to reach a better health status, as we often hypothesize. This appears to be a departure from previous findings based on the same survey but with different samples, which found that interaction with natives, as measured by immigrants’ education in China, may have a significant positive health effect on immigrants [5]. We suspect that this may be related to different sample characteristics. In the Changchun sample, a higher proportion of international migrants have organizations that can help them, such as businesses and schools. The information and resources from organizations may replace the advantages of information and resources provided by Chinese-Net, thus weakening their health utility. In contrast, Motherland-Net has a stronger explanatory power for the health changes in immigrants. People with more Motherland-Net are more likely to resist the health risks of immigration, and maintain and improve their health status in the unfamiliar society.

## 4. Discussion

Drawing on Putnam’s distinction between two types of social capital, health and social networks are viewed as dynamic processes rather than predetermined results, and these dynamic processes are constrained by both migration processes and social situations in China. There have been numerous studies suggesting a strong association between social networks, social capital, and health [24], but the focus on immigrant groups, especially those in emerging destinations, is still very limited. This is not only the neglect of an expanding group, but also the exclusion of migration processes that can have a significant impact on the construction of social networks and the health status of migrants. The data and findings of this study partially confirm the positive effect of social networks on helping migrants cope with post-migration health risks, but this effect is more reflected in Motherland-Net, which has strong connection and homogeneity. Close and trusting interpersonal relationships can provide adequate emotional support, shape healthy life patterns of immigrants, and provide long-term health promotion effects for immigrants. Although Chinese-Net is more likely to provide sufficient information, the construction of this network is more difficult for immigrant groups, especially those from developing countries and Japan and South Korea, and is further limited by the mixed residence mode. In addition, since more international migrants can receive health service assistance from their employers in the Chinese context, their dependence on Chinese-Net has decreased, and the health utility brought by Chinese-Net is also limited.

Through our analysis of the largest sample for international immigrants in Northeast China, in addition to providing new insights for research on international migrants in China, we emphasize the importance of the Chinese social context in the study of international migration. The number of immigrants in Changchun is significantly less than the cities in Pearl River Delta, such as Guangzhou and Foshan. However, it represents a different, maybe more widespread immigration pattern in Chinese society compared with cities in Pearl River Delta, where migrants are mostly from African ethnic groups, lack formal social supports, and often have legitimacy crises. The characteristics of immigrants elsewhere in China are significantly different. Under China’s practical and prudent immigration policy, people with diverse economic backgrounds and cultural concepts are welcomed by the Chinese society, but they need to undergo strict screening and are subject to continuous attention from Chinese society. This study generally contributes to the existing literature on international migration by applying an approach that tries to show the power of the Chinese social context in shaping the living conditions of immigrants to China. More importantly, we emphasize the influence of East Asian cultural concepts and the Chinese social immigration governance system on the construction of immigrant networks and health effectiveness. The inherent heterogeneity and inequality bring great complexity and flexibility to immigration networks and social capital, which leads to different social consequences.

Unfortunately, due to data limitations, we cannot satisfactorily consider the possibility of endogeneity in some of our models. In either case, the data are cross-sectional and do not support a causal argument; we can only limit our conclusions to correlations at present. For the rapidly expanding number of immigrants to China, who are only beginning to enter the academic horizon, high-quality tracking data are undoubtedly necessary in the future.

Scholars interested in continuing this line of research should consider the following. First, time is a dimension worth considering. We place equal importance on the source factor and the destination factor for first-generation immigrants. However, as first-generation immigrants stay longer and more second-generation immigrants join the international immigrant community in China, not only is the influence of destination factors likely to increase, but structural factors affecting the survival of international migrants in Chinese society may also change. Moreover, China’s reform and opening-up policy is still being constantly adjusted; it will be essential to trace the resonances between international immigrant networks and Chinese society.

Second, due to the lack of an available sampling frame, we could not achieve strict random sampling of respondents. This might have increased some inherent bias. However, as a result of China’s strict visa censorship, nearly all foreigners need to pass through the Exit and Entry Administration formalities periodically. Therefore, the time limit may be extended by further investigation. It may expand the sample size to alleviate these problems. The use of respondent-driven sampling may also be helpful for future surveys.

Third, interpersonal differences and health consequences need to be explored further through qualitative study. As our study found, the complex origins and residence status of international migrants in China reveal the differential consequences of social interaction. Since our study is based on quantitative analysis, a qualitative study on international immigrants in China involving representative individuals may be a supplement to find mediating mechanisms linking individual differences with adaptive consequences.

Finally, it is necessary to consider the potential impact of COVID-19, which may change the way people interact socially and the functioning of social capital. Some social networks may be more functional, while others may be weaker [34]. Accordingly, the foundation and functions of the international migration networks in China will also change. Data from SFRC collected after COVID-19 will enable us to explore the impact of COVID-19 on migrant networks in the future.

To provide improved answers to the aforementioned questions, we have launched a new and improved survey in 2022. This survey, with its extended time limits, has been initiated in July, with a plan to terminate it in December. Although the current survey was often interrupted due to COVID-19, presumably it aided in increasing our visitors’ representation. Furthermore, to counter the disruptions of the epidemic, we created an electronic version of the questionnaire and highly distributed it based on a respondent-driven strategy. In the questionnaire, we included more questions for measuring mindsets and behavioral patterns, pre- and post-COVID-19. At the end of the survey, we might be able to provide further insights into foreigners in China, and how their health status changed before and after the epidemic.

Furthermore, our findings have substantial implications for policy. If the goal of migrant governance is to promote their survival and development in China, it will be crucial to promote interactions between international migrants in China and the local society. Although the Chinese government continues to improve international immigration policies and laws, it pays more attention to management than to services and lacks an accurate understanding of the characteristics of immigrant groups. The practical effects of these measures have been limited or even the reverse of what was intended. On the basis of our findings, potential measures for improving the situation of international migrants in China may include providing refined service strategies for different ethnic groups, creating open mixed communities, and improving the international service level of the hospital.

## 5. Conclusions

The study investigated the construction and health change effect of different network types. The results indicated that an economic background from more developed countries improves the Chinese-Net and reduces the Motherland-Net, whereas the cultural concept of East Asia reduces the Chinese-Net and improves the Motherland-Net. A mixture of immigrants from different countries will reduce both the Motherland-Net and the Chinese-Net. Regarding the health change effect, the motherland network plays a positive role in helping immigrants to reach a better health status after coming to China, while the Chinese network has no clear positive effect on the health change in immigrants.

## Figures and Tables

**Table 1:** Multivariable linear regression model of migration network differences (N = 1624).

	Model 1	Model 2	Model 3	Model 4
VARIABLES	Chinese-Net	Chinese-Net	Motherland-Net	Motherland-Net
Control variables	Controlled	Controlled	Controlled	Controlled
High-HDI countries (base)				
Mid-HDI countries	−7.599 ***	−7.344 ***	9.769 ***	10.053 ***
	(2.482)	(2.472)	(1.948)	(1.954)
Low-HDI countries	−12.215 ***	−12.139 ***	15.797 ***	15.881 ***
	(2.051)	(2.044)	(1.350)	(1.342)
Cultural groups (0 = non-East Asian groups, 1 = East Asian groups)	−5.674 **	−6.462 **	19.191 ***	18.314 ***
	(2.688)	(2.711)	(2.065)	(2.075)
Residence form (0 = not living with many non-home immigrants, 1 = many non-home immigrants live together)		−3.580 ***		−3.983 ***
		(1.335)		(1.215)
Constant	25.931 ***	27.321 ***	17.388 ***	18.808 ***
	(6.192)	(6.219)	(5.578)	(5.616)
R-squared	0.116	0.120	0.140	0.146

Note: 1. Robust standard errors in parentheses; 2. *** *p* < 0.01, ** *p* < 0.05; 3. The control variables included survey years, age, sex, years of education, marital status, Chinese proficiency, residence duration, Engel’s coefficient, motherland immigrants in the place of residence.

**Table 2:** Robustness test of migration network differences (N = 1624).

	Model 5	Model 6	Model 7	Model 8
VARIABLES	Chinese-Net	Chinese-Net	Motherland-Net	Motherland-Net
Control variables	Controlled	Controlled	Controlled	Controlled
High-HDI countries (base)				
Mid-HDI countries	0.722 ***	0.730 ***	1.843 ***	1.869 ***
	(0.079)	(0.080)	(0.209)	(0.212)
Low-HDI countries	0.577 ***	0.578 ***	2.456 ***	2.466 ***
	(0.052)	(0.052)	(0.207)	(0.207)
Cultural groups (0 = non-East Asian groups, 1 = East Asian groups)	0.790 **	0.760 **	2.887 ***	2.765 ***
	(0.094)	(0.091)	(0.313)	(0.302)
Residence form (0 = not living with many non-home immigrants, 1 = many non-home immigrants live together)		0.841 ***		0.824 ***
		(0.055)		(0.049)
Constant	0.386 ***	0.414 ***	0.192 ***	0.206 ***
	(0.108)	(0.116)	(0.051)	(0.055)

Note: 1. Robust standard errors in parentheses; 2. *** *p* < 0.01, ** *p* < 0.05; 3. The control variables are the same as in Table 1.

## Data Availability

The data presented in this study are available upon request from the corresponding author.
